# A Pilot Study of a Synchronous Virtual Ultrasound Imaging Module for Physiotherapy Education

**DOI:** 10.1002/pri.70180

**Published:** 2026-02-26

**Authors:** Nathan J. Savage, Keiocsha R. Barnes, Ashton M. Dobbins, Price L. Barrett

**Affiliations:** ^1^ Department of Physical Therapy High Point University High Point North Carolina USA; ^2^ Department of Physical Therapy Winston‐Salem State University Winston‐Salem North Carolina USA

**Keywords:** diagnostic, education, physiotherapy, shoulder joint, ultrasonography

## Abstract

**Background and Purpose:**

This study examined the feasibility and preliminary educational impact of a single, synchronous virtual training module for developing ultrasound imaging (USI) knowledge and skills among physiotherapy students.

**Methods:**

As part of a pilot study, first‐ and second‐year physiotherapy students from one institution, all without prior USI experience, completed a baseline written examination assessing foundational USI knowledge. Second‐year students reviewed asynchronous instructional materials before participating in a one‐hour synchronous virtual training session focused on the shoulder, delivered by a faculty member board‐certified in musculoskeletal sonography. Students scanned clinically relevant shoulder structures and submitted images for blinded expert scoring based on anatomical accuracy and image quality. Following training, participants repeated the knowledge examination and completed a post‐session survey evaluating satisfaction and perceived educational value.

**Results:**

Preliminary results indicated improvements in USI knowledge and acceptable image acquisition. Baseline USI knowledge did not differ between first‐ and second‐year physiotherapy students (*N* = 34; mean difference = 0.05 points; *p* = 0.952). Second‐year students demonstrated post‐training gains in knowledge (*N* = 20; mean difference = 3.55 points; *p* < 0.001). The average composite image score was 10.4 out of 15, reflecting accurate structure identification with variable image quality. Survey responses indicated high learner engagement and perceived relevance with 90% strongly agreeing that they enjoyed learning USI, 88% found the session easy to follow, 88% viewed USI as valuable in practice, and 75% endorsed its inclusion in physiotherapy curricula.

**Discussion:**

Findings suggest that a single virtual USI training session can enhance short‐term knowledge and engagement among physiotherapy students. While results indicate promise for future curricular exploration, conclusions are limited to short‐term learning outcomes in a single pilot cohort. As USI gains recognition as a valuable point‐of‐care diagnostic tool for neuromusculoskeletal evaluation, scalable and accessible educational approaches are needed to facilitate curricular integration.

## 1 Introduction

Ultrasound imaging (USI) has emerged as a valuable adjunct to contemporary physiotherapist practice, offering high‐quality non‐invasive visualization of multiple neuromusculoskeletal structures in real‐time (APCA 2024; American Academy of MSK Ultrasound 2024; Savage, Condo, et al. 2024; Hazle et al. 2024; Page et al. 2023; Savage et al. 2023; Smith et al. 2022; Ellis et al. 2020; Whittaker et al. 2019; Kostopoulos and Rawat 2017; Imaging Special Interest Group et al. 2015). The growing use of USI as a point‐of‐care diagnostic tool in physiotherapist practice reflects its diagnostic utility and versatility but also its capacity to support movement‐based assessment, guide targeted interventions, and reinforce clinical reasoning through visual biofeedback (Savage, Bell‐Linnear, et al. 2024; Savage, George, et al. 2025; Savage and McKell 2024; Rawat 2020; Kostopoulos and Rawat 2019). In contrast to other imaging modalities, USI is portable, cost‐effective, and safe being free of ionizing radiation—qualities that make it particularly suited for point‐of‐care integration into physiotherapist practice (Rawat 2020; Jacobson 2018).

Despite its clinical relevance, USI exposure in physiotherapy curricula remains inconsistent. Virtual platforms may provide scalable solutions to foundational training, but their educational impact among learners remains unclear. Contemporary physiotherapist practice is increasingly focused on training autonomous, first‐contact primary care providers capable of synthesizing advanced neuromusculoskeletal evaluations (Savage, Nielsen, et al. 2025; APTA American Physical Therapy Association 2024; Baumbach et al. 2024; Henning et al. 2024; O’Bright and Peterson 2024; Budtz et al. 2022; Clark et al. 2022; Bodenheimer et al. 2021; Jackson et al. 2021; Bornhöft et al. 2019; Keil et al. 2019; Downie et al. 2019). Within this evolving model, diagnostic imaging literacy—specifically USI competence—is increasingly viewed as an essential component of advanced practice readiness.

Integration of USI in physiotherapy curricula could accelerate competence, foster interdisciplinary collaboration, and expand the role of physiotherapists in delivering high‐value care. However, logistical barriers including limited faculty expertise, lack of equipment, and curricular crowding have slowed widespread adoption in medical and physiotherapy education (Savage, Condo, et al. 2024). Accordingly, there is an urgent need for scalable educational models that introduce foundational USI skills efficiently within existing curricular structures. Prior studies have demonstrated that short, focused educational modules can enhance knowledge and psychomotor skills in imaging and other clinical competencies (Cowan et al. 2025; Lake et al. 2024; Neubauer et al. 2023; Brown et al. 2022; Huang et al. 2022; Bowers et al. 2021; Nicholas et al. 2021; Cho and Reckelhoff 2019; Tarique et al. 2018; Woods et al. 2018; So et al. 2017; Rempell et al. 2016; Dinh et al. 2015). However, the feasibility and effectiveness of virtual training modules specifically targeting the performance of USI among physiotherapy students remains poorly studied, despite the growth of virtual pedagogy in health professions education during the handling of the COVID‐19 pandemic (Wilcha 2020).

The purpose of this pilot study was to explore the feasibility and preliminary educational impact of a single synchronous virtual training module among physiotherapy students at one institution. The shoulder region was selected because of its clinical relevance, complex anatomy, and technical difficulty in sonographic examination (Kostopoulos and Rawat 2017; Rawat 2020; Jacobson 2018; Ingwersen et al. 2016; Bailey et al. 2015). We anticipated that students would demonstrate measurable learning gains and have positive perceptions of USI, supporting the feasibility of this instructional approach.

## 2 Methods

This pilot study used a mixed design to assess feasibility, learner engagement, and preliminary learning outcomes to evaluate changes in USI knowledge and psychomotor skill performance among physiotherapy students. The primary outcome measures were (1) basic USI knowledge examination scores—comparing first‐ and second‐year physiotherapy students and pre‐/post‐training scores within the second‐year cohort—and (2) image performance scores reflecting the anatomical accuracy and quality of student‐generated USI images reviewed by a blinded expert evaluator.

### 2.1 Participants

Eligible participants were current first‐ and second‐year physiotherapy students actively enrolled in coursework, with no prior didactic or practical experience performing USI and no self‐reported shoulder pain or pathology that could limit participation. Participants were recruited through classroom announcements and email communication.

Participants provided written informed consent prior to data collection, and confidentiality of all personal and health information was maintained through coded identifiers and password‐protected data storage. Following consent, participants completed a demographic questionnaire and a baseline examination assessing basic USI knowledge (Appendix A). The 12‐item knowledge examination was developed by a content expert in musculoskeletal USI education and reviewed by three coinvestigators for content validity and clarity. The items covered core musculoskeletal USI principles, transducer handling, echotexture interpretation, and anatomical identification. Although this instrument has not undergone formal psychometric validation, its internal structure and relevance to the module's learning objectives support its use as an exploratory assessment in this pilot study.

Because of scheduling constraints, only second‐year students participated in the synchronous virtual training session and subsequent post‐test assessment. First‐year students completed the baseline knowledge test only to serve as a comparative reference group for pre‐training USI knowledge.

### 2.2 Pre‐Training

**FIGURE 1:**
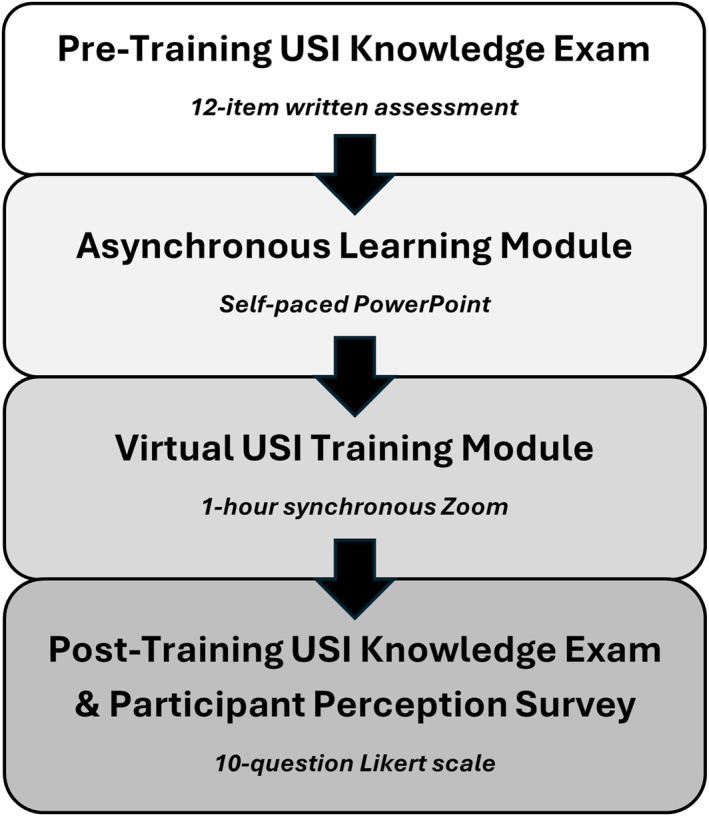
Study workflow for the educational pilot. Participants completed asynchronous pre‐training, a synchronous virtual ultrasound imaging session, and immediate post‐training knowledge and perception assessments.

Prior to the synchronous session, second‐year students were provided with a self‐paced PowerPoint module containing core USI principles, transducer orientation conventions, and labeled images and videos illustrating specific shoulder scanning procedures and target anatomical structures (Figure 1).

### 2.3 Training Procedures

Based on the availability of USI devices, training sessions could accommodate a maximum of 5 pairings (10 participants total). During the one‐hour virtual USI training session, students were paired as “therapist” and “patient.” Each pair was assigned a 15 MHz Clarius handheld linear array transducer (L15HD3, Clarius; Vancouver, BC, Canada) connected to an iPad Pro 10.5‐inch tablet (Apple; Cupertino, CA, USA) to acquire all study‐related images. Under real‐time virtual guidance, students alternated scanning clinically relevant shoulder structures on their partners as directed by the instructor. The instructor is a physical therapist and full‐time faculty member board‐certified in musculoskeletal sonography with more than 8 years of clinical, teaching, and research experience using USI, including virtual training modules.

Virtual instruction was delivered via a secure Zoom videoconferencing platform (Zoom Video Communications; San Jose, CA, USA) projected on a large display in a regular classroom. The instructor performed all scanning demonstrations on a co‐investigator positioned facing the webcam with the right shoulder exposed for imaging demonstration.

To familiarize students with the handheld device and optimize initial image acquisition, participants were first guided through instructor‐directed scanning of anterior wrist structures—a region selected for its distinct visualization of bone, tendon, nerve, and vascular landmarks. This brief orientation facilitated comprehension of echogenic pattern interpretation and real‐time image optimization.

Following orientation, each student scanned their partner's right shoulder while sitting. The instructor provided step‐by‐step verbal instruction and live USI demonstrations for each of the six target shoulder structures: (1) long‐head biceps tendon in short‐ and long‐axis views, (2) subscapularis tendon and insertion in long‐axis view, (3) acromioclavicular joint in coronal‐oblique view, and (4–6) supraspinatus tendon and insertion in short‐ and long‐axis views including the rotator interval, which displays a short‐axis view of the long‐head biceps tendon (Kostopoulos and Rawat 2017; Rawat 2020; Jacobson 2018).

After each student completed the instructor‐guided scanning sequence, they independently repeated the process on the same shoulder and in the same order as they trained (i.e., first student finished scanning with instructor guidance, followed by the second student; first student completed independent scanning, followed by the second student). Students were instructed to capture optimized images of each target structure for later review and scoring emphasizing probe stability, image centering, and adjustment of gain and depth parameters to enhance image clarity.

Two coinvestigators provided in‐person logistical support (e.g., equipment set up and troubleshooting) and monitored adherence to study procedures but did not provide any instructional guidance or feedback regarding transducer placement, structure identification, or image optimization.

### 2.4 Post‐Training

Following the USI virtual training session, participants were asked to complete the same 12‐item examination assessing basic USI knowledge that they took at baseline (Appendix A). Additionally, participants were asked to complete a 5‐point Likert scale survey (i.e., “Strongly agree,” “Agree,” “Neither agree nor disagree,” “Disagree,” and “Strongly disagree”) about their experience with the virtual training session and some questions about the future of USI education in physiotherapy programs.

### 2.5 Image Review and Scoring

Each student pair's iPad was wirelessly linked to the Clarius application for acquisition and storage of USI images. Captured images were labeled with unique participant identifiers, de‐identified, and exported as password‐protected files for blind evaluation.

A single blinded expert reviewer—Registered in Musculoskeletal sonography with more than 8 years of experience performing, teaching, and publishing in USI—assessed all submitted images. Images were randomized prior to review to prevent recognition of specific participants or pairings.

Each of the six target images was scored using a structured Likert‐type scale (Table 1). Scoring criteria included (1) location accuracy, defined by visualization of correct bony landmarks and (2) image quality and clarity, defined by the resolution and delineation of the target structure (e.g., tendon borders, joint line).

**TABLE 1:** Scoring rubric for ultrasound image acquisition performance.

Structure & view	Example image	Transducer position	Scoring
Long head biceps tendon in short axis	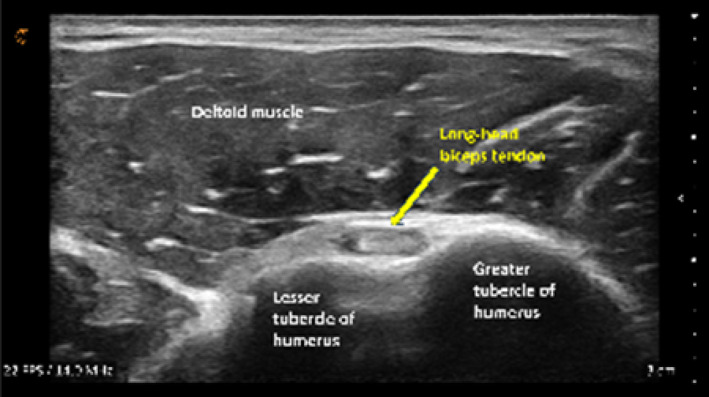	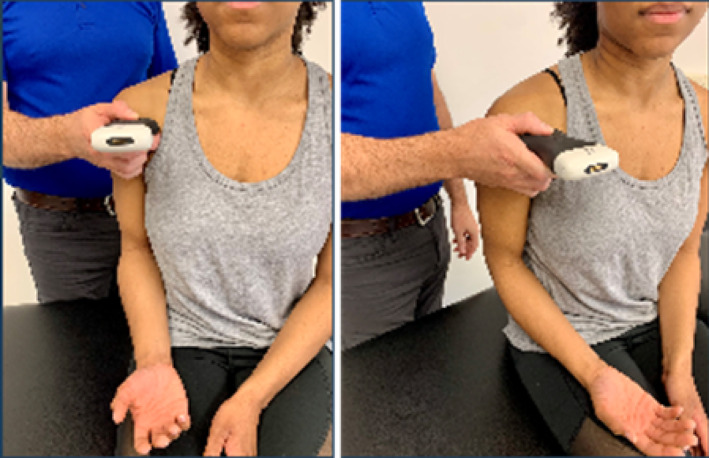	2, 1, or 0
Long head biceps tendon in long axis view^a^	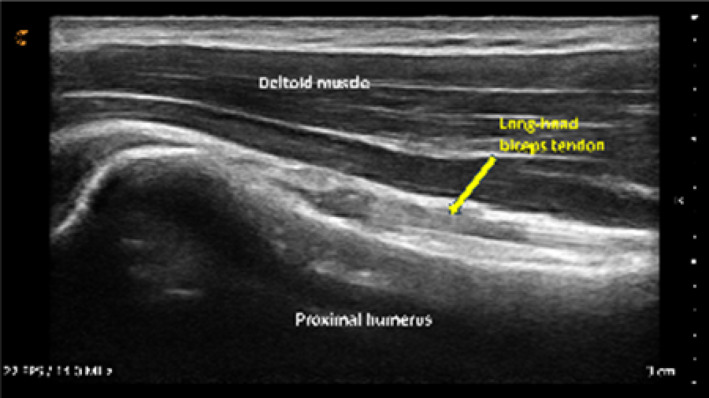	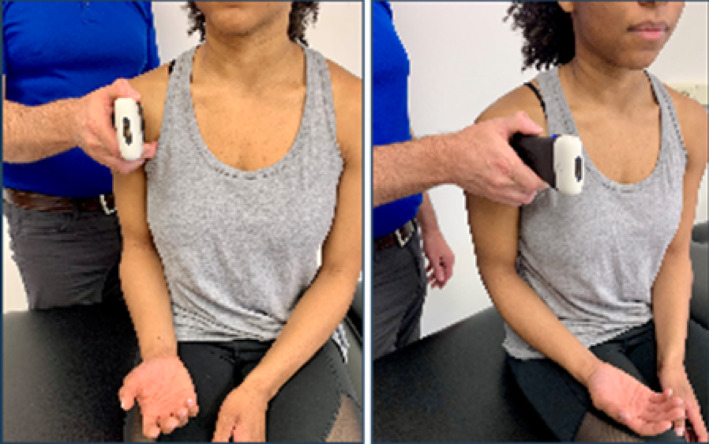	3, 2, or 0
Subscapularis tendon & insertion in long axis view	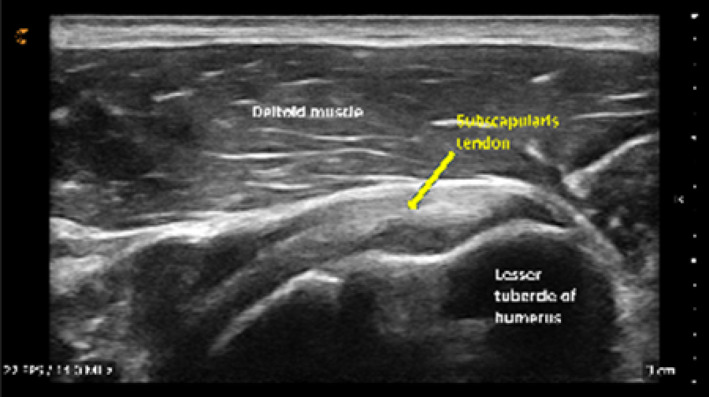	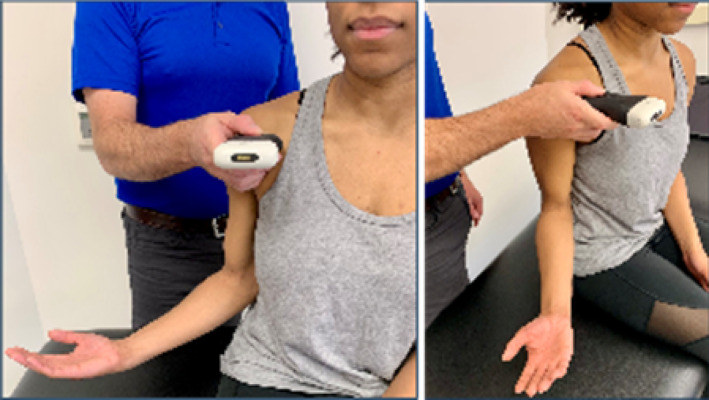	2, 1, or 0
Acromioclavicular joint in coronal‐oblique axis view^a^	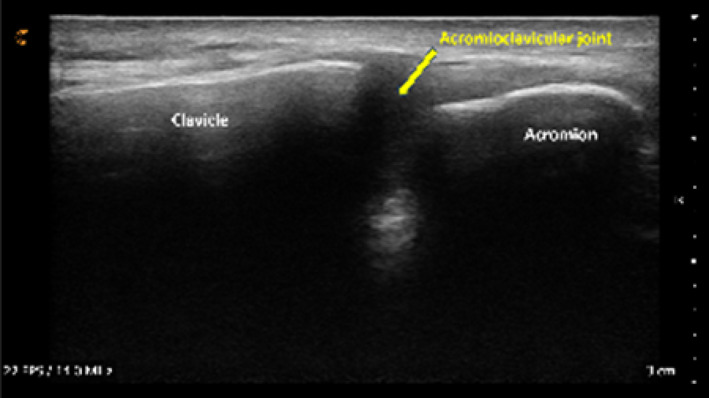	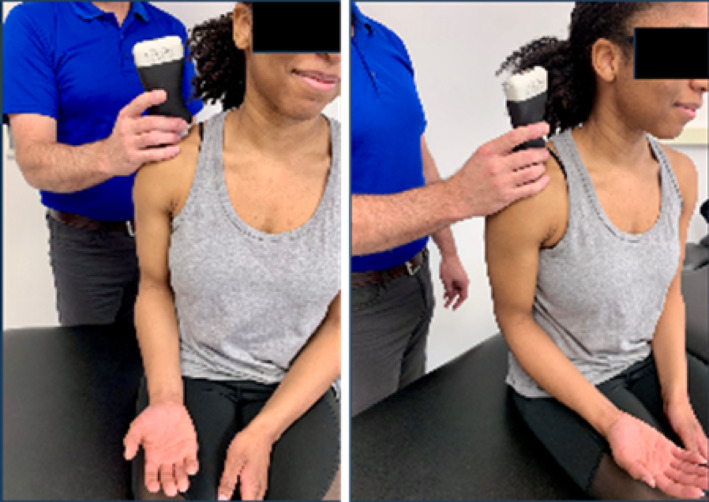	3, 2, or 0
Supraspinatus tendon & insertion in long axis view	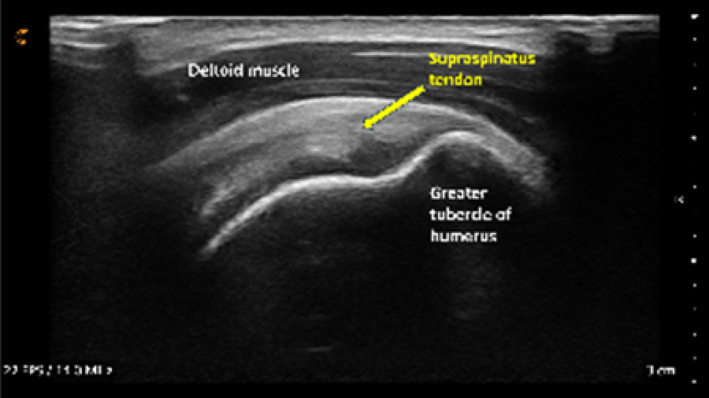	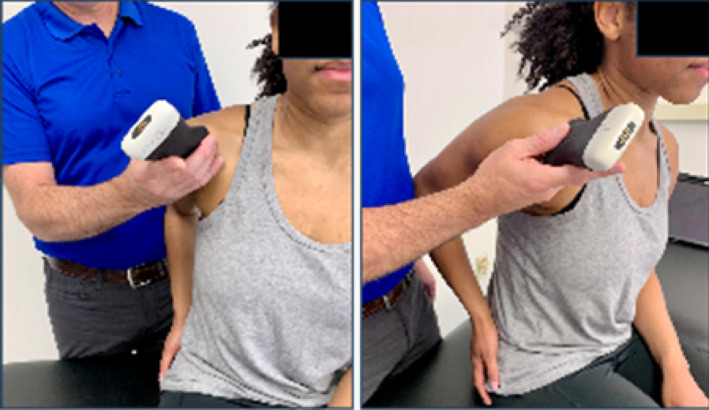	2, 1, or 0
Supraspinatus tendon & rotator interval in short axis view^a^	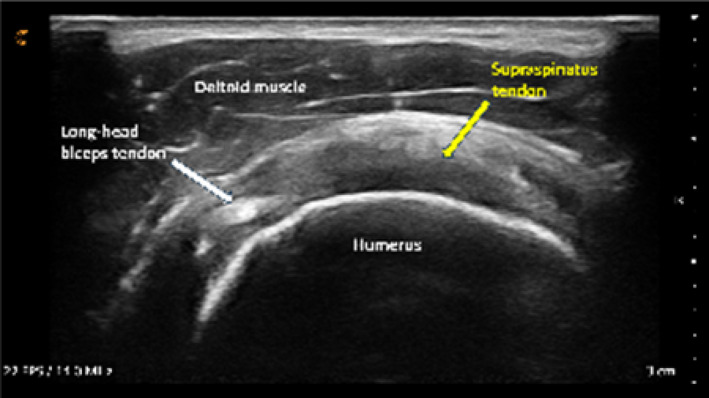	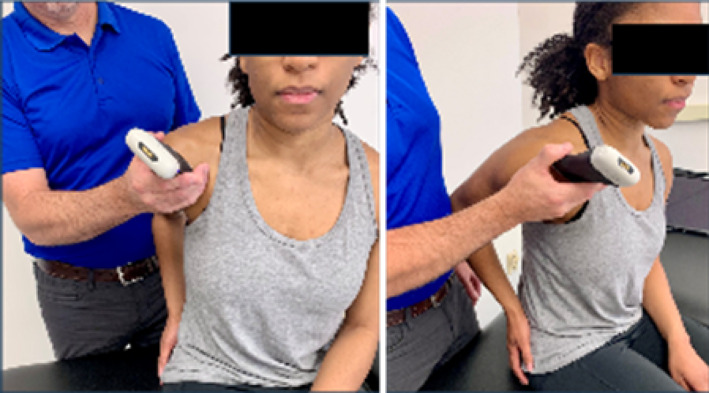	3, 2, or 0

*Note:* Each image was scored for anatomic accuracy and image quality, with higher weights applied to more technically challenging structures. Total composite score range: 0–15.

^a^ Considered more challenging images to obtain.

The image‐scoring rubric was developed by a content expert in musculoskeletal USI education and reviewed by three coinvestigators for content validity and clarity. The scoring rubric was adapted from previously published USI educational studies evaluating anatomical accuracy and image quality in novice learners (Hoppmann et al. 2022, 2015). Scoring criteria emphasized (1) correct anatomic localization, verified by identification of key bony or tendinous landmarks, and (2) overall image quality and clarity, reflecting appropriate gain, depth, and centering. Higher‐difficulty structures (e.g., long head biceps tendon viewed in long axis, acromioclavicular joint, and supraspinatus tendon viewed in short axis) were weighted proportionally greater to reflect their increased technical challenge. The composite score (0–15 points) provided a global measure of technical performance.

Prior to scoring, the blinded expert rater reviewed a standardized set of reference images and calibration examples from coinvestigators and pilot participants to establish internal consistency. Although a single expert rater was used, reliability was strengthened through this pre‐scoring and calibration process and the use of explicit and established scoring criteria.

Three of the six images—subscapularis, supraspinatus long‐axis, and acromioclavicular joint—were classified a priori as higher‐difficulty structures and assigned proportionally greater possible scores. The composite score ranged from 0 (no correct structures visualized) to 15 (all structures visualized with high‐quality images).

This study was reviewed and approved by the Institutional Review Board at Winston‐Salem State University (IRB‐FY2025‐5). All participants provided informed consent prior to enrollment. The study adhered to the ethical principles outlined in the Declaration of Helsinki.

### 2.6 Statistical Analysis

All statistical analyses were performed using IBM SPSS Statistics, version 29.0.1.0 (Armonk, NY, USA). Descriptive statistics were used to summarize demographic characteristics, baseline and post‐training USI knowledge scores, image scores, and post‐training survey responses.

Independent samples *t*‐tests compared baseline USI knowledge between first‐ and second‐year students, and paired samples *t*‐tests evaluated within‐group pre‐/post‐training changes among second‐year students. Effect sizes (Hedges' *g*) were calculated to quantify the magnitude of observed differences.

Pearson's correlation coefficients were computed to examine associations between pre‐training engagement (e.g., review of asynchronous materials), USI knowledge scores, and image quality scores. Alpha was set at 0.05 for all analyses, and assumptions of normality and homogeneity of variance were verified prior to inferential testing. Descriptive statistics, 95% confidence intervals, and effect sizes accompany all reported *p*‐values.

Reliability analysis for the image‐scoring system was not conducted because of the use of a single blinded expert rater; however, procedural consistency was maintained through prior calibration and adherence to standardized scoring criteria.

## 3 Results

### 3.1 Participant Characteristics

Thirty‐four physiotherapy students participated in this pilot study (10 first‐year, 24 second‐year). The synchronous virtual USI training session and post‐training survey were completed by all 20 participating second‐year students (Table 2).

**TABLE 2:** Characteristics of first‐ and second‐year physiotherapy student participants.

Characteristics	All participants	USI participants
	(*N* = 34)	(*N* = 20)
	Count (%)	
Program year		
First	10 (29%)	—
Second	24 (71%)	20 (100%)
Sex		
Male	12 (35%)	8 (40%)
Female	22 (65%)	12 (60%)
Race/ethnicity		
Asian	3 (9%)	2 (10%)
Black	14 (41%)	8 (40%)
Hawaiian	1 (3%)	1 (5%)
Bi/multi‐racial	15 (44%)	—
White	1 (3%)	9 (45%)
Dominate hand		
Right	31 (91%)	17 (85%)
Left	3 (9%)	3 (15%)

**Characteristics**	**Mean ± SD**	
Age	25.3 ± 2.6	25.6 ± 2.9
Basic USI knowledge score		
Baseline	4.24 ± 2.1	4.40 ± 1.8
Post‐training	—	7.95 ± 2.4

Abbreviation: SD, standard deviation.

Among the second‐year students completing the USI training, 14 out of 20 (70%) reported reviewing the asynchronous pre‐training materials prior to the scanning session, whereas 6 (30%) did not. Of those who reviewed the materials, 11 students (79%) reported viewing them once, while 3 students (21%) reviewed them between two and four times.

All participants successfully completed both the scanning and post‐test components without adverse events or equipment issues.

### 3.2 Basic USI Knowledge

The mean baseline written examination score for all students was 35%, with 11 out of the 12 questions scoring ≤ 50%. This finding indicates limited prior exposure to fundamental USI concepts among physiotherapy students.

Independent samples *t*‐tests revealed no significant difference in baseline USI knowledge scores between first‐ and second‐year physiotherapy students (*N* = 34; mean difference = 0.05 points; [95% CI, −1.66 to 1.56; *p* = 0.952; *g* = 0.02]), indicating comparable knowledge levels across cohorts.

**FIGURE 2:**
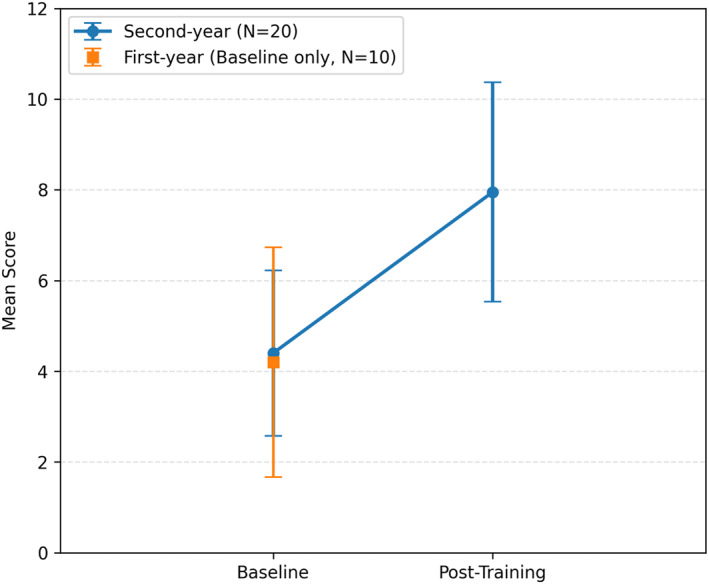
Basic ultrasound imaging knowledge examination scores among first‐year (baseline only) and second‐year physiotherapy students.

Among second‐year students who completed the USI training, the post‐training knowledge score increased by a mean of 3.55 points (95% CI, 2.39 to 4.70; *p* < 0.001; *g* = 1.39), representing a large effect size and a 29% mean performance improvement (Figure 2).

At post‐test, 11 out of the 12 examination questions scored ≥ 50%, with the only exception being question 8. Half of the test items (6 out of 12) demonstrated ≥ 100% improvement in raw scores, indicating broad conceptual gains across multiple content areas.

Engagement with pre‐training materials was not meaningfully associated with USI knowledge or imaging performance outcomes (all *r* values < 0.20 and nonsignificant).

### 3.3 Image Quality

The mean composite image score for all second‐year physiotherapy students was 10.4 out of 15 points (69%), with most individual images scoring ≥ 75%. The median image score was slightly higher at 11.5 out of 15 (77%), and one student achieved a perfect score. These results indicate that participants demonstrated short‐term success in localizing shoulder structures following virtual instruction though image quality varied moderately among students.

**FIGURE 3:**
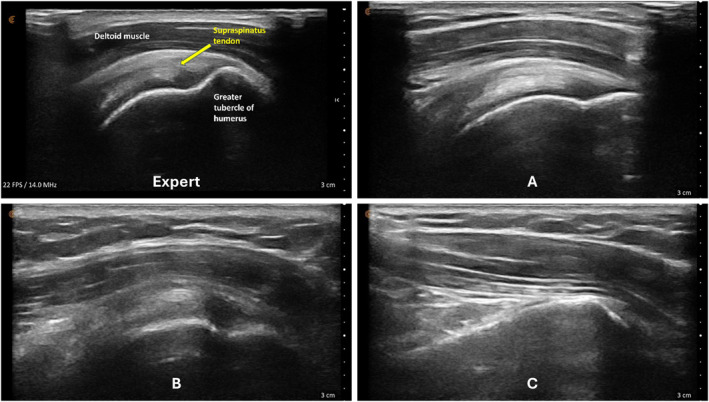
Supraspinatus tendon and insertion onto the greater tubercle of humerus. Expert image (Expert, *Top Left*), student image receiving full points (A, *Top Right*), student image receiving partial points (B, *Bottom Left*), and student image receiving no points (C, *Bottom Right*).

The most challenging structures to visualize were the short‐ and long‐axis views of the long‐head biceps tendon (Table 3). These scans typically require precise transducer alignment and depth adjustment, contributing to their lower image quality scores. Representative examples of supraspinatus tendon images demonstrating variable quality are shown in Figure 3.

**TABLE 3:** Post‐training ultrasound image performance by structure.

Target structure	Mean ± SD	Percentage	Median (range)
Long head biceps tendon	1.15 ± 0.75	58	1.00 (0–2)
Long head biceps tendon	1.70 ± 1.08	57	2.00 (0–3)
Subscapularis tendon & insertion	1.50 ± 0.51	75	1.50 (1–2)
Acromioclavicular joint	2.25 ± 1.07	75	3.00 (0–3)
Supraspinatus tendon & insertion	1.55 ± 0.69	78	2.00 (0–2)
Supraspinatus tendon & rotator interval	2.25 ± 0.72	75	2.00 (0–3)
Cumulative score	10.4 ± 0.80	69	11.5 (77%)

*Note:* Scores reflect blinded expert ratings using standardized rubric.

Abbreviation: SD, standard deviation.

No significant correlations emerged between pre‐training material engagement, USI knowledge test performance, and image scores. A small but statistically significant positive correlation was identified between post‐training question 12 (focused on the short‐axis view of the long‐head biceps tendon) and image 1 scores of the same structure (*r* = 0.447, 95% CI 0.006–0.743). This suggests limited alignment between theoretical knowledge and practical image acquisition for this specific anatomical region. All other correlations between image‐specific questions and corresponding image scores were nonsignificant (*r* = 0.000–0.385), indicating that written test performance and image quality were largely independent skill domains.

Overall, second‐year students demonstrated the ability to obtain anatomically accurate images of major shoulder structures following brief virtual instruction, with variability primarily reflecting differences in sonographic fine‐motor control and real‐time probe manipulation.

### 3.4 Post‐Training Survey

Survey responses were received an average of 10 days after the USI training session (range, 7–26 days). Survey items were evenly divided between questions regarding engagement with pre‐training materials and perceptions of the virtual training experience itself.

The mean overall survey score among second‐year students was 1.53 out of 2 points (77%), with 7 out of 10 items scoring ≥ 75%. These results reflect consistently positive perceptions of the instructional experience and perceived clinical relevance of USI.

Students overwhelmingly reported that they enjoyed learning about and performing USI, and most expressed enthusiasm for expanding their training to include additional body regions. Several respondents indicated interest in a dedicated elective course or longitudinal USI module within the physiotherapy curriculum, highlighting a strong appetite for continued exposure.

Additionally, students agreed that USI represents a valuable tool for physiotherapist practice and should be formally included in physiotherapy education (Table 4). Collectively, these findings suggest that brief, focused virtual instruction not only improves foundational USI skills but also fosters positive attitudes toward integrating USI into clinical reasoning and patient care.

**TABLE 4:** Post‐training learners' perceptions and satisfaction ratings.

	Survey question	Mean ± SD (range)	Percentage
Training	I enjoyed learning about ultrasound imaging	1.80 ± 0.41 (1–2)	90
	I enjoyed performing ultrasound imaging of the shoulder region	1.85 ± 0.37 (1–2)	93
	The pretraining course materials were easy to understand	1.25 ± 0.72 (0–2)	63
	It was easy to follow along during the in‐person shoulder scanning session	1.75 ± 0.44 (1–2)	88
	I was able to locate most of the shoulder structures covered during the in‐person scanning session	1.60 ± 0.50 (1–2)	80
Future	I would like to learn how to perform ultrasound imaging of other body regions	1.70 ± 0.47 (1–2)	85
	I think ultrasound imaging is a valuable tool for physiotherapist practice	1.75 ± 0.44 (1–2)	88
	I think the performance of ultrasound imaging should be taught in physiotherapy programs	1.50 ± 0.51 (1–2)	75
	I would take an elective course in ultrasound imaging if it was offered by my physiotherapy program	1.35 ± 0.49 (1–2)	68
	I plan to seek additional training in ultrasound imaging after I graduate	0.75 ± 0.85 (−1 to 2)	38
	Cumulative mean	1.53 ± 0.52	77

*Note:* Items were scored on a 5‐point Likert scale (1 = strongly disagree, 5 = strongly agree).

Abbreviation: SD, standard deviation.

## 4 Discussion

### 4.1 Key Findings

This pilot study explored the educational impact of a single synchronous virtual USI training session among physiotherapy students and found measurable short‐term knowledge gains and basic skill acquisition along with positive learner perceptions. While the composite image scores for second‐year physiotherapy students (mean 69%, median 77%) were positive findings indicating success in localizing shoulder structures following virtual instruction, these values should not be equated with clinical competence. Mastery of this clinical skill requires additional supervised training in the performance and interpretation of USI. These findings highlight the potential of virtual USI instruction and the feasibility of this educational approach. Observed learning gains were accompanied by large effect sizes and narrow confidence intervals, reinforcing the stability of these pilot findings despite the limited sample size from a single institution.

The observed gains in basic USI knowledge, the ability to accurately obtain images of clinically relevant shoulder structures, and the high levels of learner satisfaction collectively indicate that virtual interactive instruction may serve as a viable and scalable approach for integrating USI education into physiotherapy curricula. This aligns with evidence from medical and allied health education demonstrating that concise, targeted modules can support early skill development relevant to clinical practice (Cowan et al. 2025; Brown et al. 2022; Woods et al. 2018; Kitagawa et al. 2022; Bitterman, Oh‐Park, et al. 2020; Mehta et al. 2018; Royer 2016).

### 4.2 Educational Relevance

These results are particularly meaningful given the increasing emphasis on diagnostic reasoning and imaging competencies within contemporary physiotherapy education (Savage, Condo, et al. 2024; Hazle et al. 2024; Whittaker et al. 2019; Hayward et al. 2022). National survey data indicate that, although most physiotherapy programs recognize the value of USI training, fewer than 10 instructional hours are typically provided. Adoption remains constrained by limited faculty expertise, high equipment costs, and curricular overcrowding (Savage, Condo, et al. 2024). This resource gap has created a disconnect between professional interest and practical implementation, leaving most physiotherapy students with minimal exposure to imaging technology during training.

Similarly, while medical schools have accelerated USI integration, only 62% report formal curricular inclusion, and barriers remain largely centered on resource limitations and instructor availability. Virtual training may represent a promising and accessible delivery model for introducing USI concepts and training in resource‐limited settings; however, broader curricular integration requires further investigation across institutions and learner cohorts (Neubauer et al. 2023; Nicholas et al. 2021; Bitterman, Lew, et al. 2020).

Currently, USI is not a required competency in physiotherapy curricula in the United States (CAPTE 2023). Moreover, limited empirical evidence exists on optimal pedagogical approaches for teaching USI in physiotherapy programs, and no consensus standard defines the appropriate depth or breadth of training (Royer 2016). Nevertheless, evidence from medical education supports the idea that early and repeated exposure to USI enhances clinical competency and reinforces anatomical understanding and physical examination skills (Cowan et al. 2025). For physiotherapy, such reinforcement may be particularly valuable as the discipline's diagnostic process relies heavily on anatomical palpation, movement analysis, and tissue differentiation—skills that are directly complemented by real‐time USI visualization.

Such eventual competencies can enhance interprofessional collaboration and contribute to value‐based, cost‐effective care models. A recent qualitative survey examining diagnostic USI use among physiotherapists holding physician‐level musculoskeletal sonography certification (Registered in Musculoskeletal Sonography [https://www.apca.org/msk‐physical‐therapists/]) found that these specialists performed USI on nearly one‐third of their patients, and that the imaging information directly influenced the patient's diagnosis and medical management in every case (Hayward et al. 2022). These findings underscore the growing clinical relevance of USI and the need to prepare future physiotherapists for its integration into routine practice.

Technological advances, such as handheld USI transducers, have made integration more feasible and cost‐effective (Falkowski et al. 2020), yet many health professions programs continue to face a shortage of qualified instructors. When expert faculty are unavailable, virtual instruction may offer a temporary or introductory mechanism for exposing students to USI concepts and training. Further work is needed to determine whether such experiences translate into sustainable learning gains and skill retention (Lake et al. 2024).

### 4.3 Limitations

This pilot study was limited by the small sample size at a single institution with no control or comparison group. Outcomes were short‐term, with no assessment of knowledge or skill retention or performance in an authentic clinical environment. The post‐training survey completed on average 10 days following the virtual training session could introduce recall bias.

While the development of imaging competencies aligns with the physiotherapy profession's evolving diagnostic role, this pilot's scope was limited to short‐term educational outcomes. The findings should not be interpreted as evidence of clinical competency or readiness for independent imaging practice. Imaging proficiency allows physiotherapists to better triage neuromusculoskeletal conditions, guide conservative interventions, and identify cases requiring medical referral—all hallmarks of primary care physiotherapist practice.

A key limitation of this pilot study was the absence of formal validation or reliability testing for the knowledge assessment and image‐scoring rubric. Although both tools were developed by an education expert in musculoskeletal USI and similar tools have been used before in studies evaluating USI learning, their psychometric properties remain unverified.

### 4.4 Future Directions

Future research should employ multi‐institutional designs with validated assessment tools, randomized control conditions, and longitudinal follow‐up to examine knowledge and skill retention and transfer to clinical practice. Comparative research across virtual, in‐person, and hybrid formats could identify efficient, evidence‐informed approaches to integrating USI education within physiotherapy curricula.

## 5 Implications for Physiotherapy Practice

The findings of this pilot study support the short‐term educational feasibility of synchronous virtual USI training for facilitating initial learning in both cognitive and psychomotor domains. Moreover, USI training was perceived by physiotherapy students as a highly valuable skill for their education and future clinical practice, reflecting strong learner engagement and perceived professional relevance. This aligns with the broader shift within the profession toward evidence‐informed diagnostic practice and patient‐centered care.

As the physiotherapy profession continues to evolve toward autonomous, first‐contact primary care roles, the integration of point‐of‐care diagnostic technologies such as USI becomes increasingly essential within physiotherapy education (Savage, Nielsen, et al. 2025; O’Bright and Peterson 2024). Integrating USI‐based competencies has the potential to enhance clinical reasoning, diagnostic precision, and reinforce the physiotherapist's role as a valuable interdisciplinary healthcare provider.

Virtual and hybrid training models are feasible and cost‐efficient pedagogical innovations that mirror trends across health profession education, where simulation‐based and technology‐enhanced learning are used to democratize access to specialized skills training. For physiotherapy, this approach provides a mechanism to expand USI education without displacing existing content, preserving curricular balance while advancing diagnostic capability.

The next critical step for the physiotherapy profession is to define consensus‐based minimal competencies for USI within educational curricula. Developing such competencies will ensure that graduates enter practice prepared to integrate USI responsibly and effectively, strengthening the diagnostic precision, safety, and value of physiotherapist practice.

## Funding

The authors have nothing to report.

## Ethics Statement

All study procedures were approved for use in human subjects by the Institutional Review Board at Winston‐Salem State University (IRB‐FY2025‐5).

## Consent

Participants provided written informed consent prior to data collection.

## Conflicts of Interest

The authors declare no conflicts of interest.

## Data Availability

Data are available upon request.

## Appendix A

This 12‐item written knowledge test was developed by a faculty expert to assess foundational ultrasound imaging principles, image interpretation, and anatomical recognition. Items were reviewed for face and content validity prior to use.

1. Which of the following best describes point‐of‐care ultrasound imaging?
  A. Diagnostic laboratory procedure
  B. Extension of the clinical examination
  C. Valuable but expensive modality
  D. Valuable but with known risks
2. Which of the following transducers will optimize visualization of superficially located structures?
  A. High frequency linear
  B. Medium frequency linear
  C. Low frequency curvilinear
  D. Cross frequency phasic
3. Which of the following transducer movements is most likely to reduce anisotropy when visualizing the long head biceps tendon in the short axis?
  A. Sliding
  B. Heel‐toe
  C. Tilt or toggle
  D. Rotation
4. Which of the following structures will appear as speckled or honeycomb when viewed with ultrasound imaging in the short axis?
  A. Normal ligament
  B. Normal nerve
  C. Normal vessel
  D. Normal tendon
5. Which of the following structures will appear hyperechoic when viewed using ultrasound imaging?
  A. Normal vessel
  B. Normal nerve
  C. Normal muscle
  D. Normal tendon
6. Which of the following best describes the appearance of a normal vessel when viewed using ultrasound imaging?
  A. Anechoic
  B. Hypoechoic
  C. Isoechoic
  D. Hyperechoic
7. Which of the following best describes the appearance of tendinosis/tendinopathy when viewed using ultrasound imaging?
  A. Hypoechoic and thinned
  B. Hyperechoic and thinned
  C. Hypoechoic and thickened
  D. Hyperechoic and thickened
8. Which of the following transducer positions is most appropriate to visualize the subscapularis tendon in the long axis?
  A. Horizontal
  B. Vertical
  C. Frontal plane
  D. Oblique plane
9. Which of the following shoulder muscles is depicted in this image?
  A. Deltoid
  B. Biceps
  C. Supraspinatus
  D. Subscapularis
10. Which of the following shoulder structures is depicted in this image?
  A. Proximal humerus bone
  B. Glenohumeral joint
  C. Long head of biceps tendon
  D. Acromioclavicular joint
11. Which of the following best describes this image?
  A. Long axis view of the subscapularis tendon
  B. Long axis view of the supraspinatus tendon
  C. Short axis view of the subscapularis tendon
  D. Short axis view of the supraspinatus tendon
12. Which of the following best describes this image?
  A. Short axis view of the long head biceps tendon
  B. Long axis view of the long head biceps tendon
  C. Short axis view of the subscapularis tendon
  D. Long axis view of the supraspinatus tendon
